# A transcriptomic analysis of sugarcane response to *Leifsonia xyli* subsp. *xyli* infection

**DOI:** 10.1371/journal.pone.0245613

**Published:** 2021-02-02

**Authors:** Kai Zhu, Li-Tao Yang, Cheng-Xi Li, Prakash Lakshmanan, Yong-Xiu Xing, Yang-Rui Li

**Affiliations:** 1 College of Agriculture, Guangxi University, Nanning, Guangxi, China; 2 Ministry of Agriculture Key Laboratory of Sugarcane Biotechnology and Genetic Improvement (Guangxi), Guangxi Key Laboratory of Sugarcane Genetic Improvement, Sugarcane Research Center, Chinese Academy of Agricultural Sciences, Sugarcane Research Institute, Guangxi Academy of Agricultural Sciences, Nanning, Guangxi, China; 3 Interdisciplinary Center for Agriculture Green Development in Yangtze River Basin, College of Resources and Environment, Southwest University, Chongqing, China; 4 Queensland Alliance for Agriculture and Food Innovation, The University of Queensland, St Lucia, Queensland, Australia; Fujian Agriculture and Forestry University, CHINA

## Abstract

Sugarcane ratoon stunting disease (RSD) caused by *Leifsonia xyli* subsp. *xyli* (*Lxx*) is a common destructive disease that occurs around the world. *Lxx* is an obligate pathogen of sugarcane, and previous studies have reported some physiological responses of RSD-affected sugarcane. However, the molecular understanding of sugarcane response to *Lxx* infection remains unclear. In the present study, transcriptomes of healthy and *Lxx*-infected sugarcane stalks and leaves were studied to gain more insights into the gene activity in sugarcane in response to *Lxx* infection. RNA-Seq analysis of healthy and diseased plants transcriptomes identified 107,750 unigenes. Analysis of these unigenes showed a large number of differentially expressed genes (DEGs) occurring mostly in leaves of infected plants. Sugarcane responds to *Lxx* infection mainly via alteration of metabolic pathways such as photosynthesis, phytohormone biosynthesis, phytohormone action-mediated regulation, and plant-pathogen interactions. It was also found that cell wall defense pathways and protein phosphorylation/dephosphorylation pathways may play important roles in *Lxx* pathogeneis. In *Lxx*-infected plants, significant inhibition in photosynthetic processes through large number of differentially expressed genes involved in energy capture, energy metabolism and chloroplast structure. Also, *Lxx* infection caused down-regulation of gibberellin response through an increased activity of DELLA and down-regulation of GID1 proteins. This alteration in gibberellic acid response combined with the inhibition of photosynthetic processes may account for the majority of growth retardation occurring in RSD-affected plants. A number of genes associated with plant-pathogen interactions were also differentially expressed in *Lxx*-infected plants. These include those involved in secondary metabolite biosynthesis, protein phosphorylation/dephosphorylation, cell wall biosynthesis, and phagosomes, implicating an active defense response to *Lxx* infection. Considering the fact that RSD occurs worldwide and a significant cause of sugarcane productivity, a better understanding of *Lxx* resistance-related processes may help develop tools and technologies for producing RSD-resistant sugarcane varieties through conventional and/or molecular breeding.

## Introduction

Ratoon stunting disease (RSD) was first discovered in Queensland, Australia in 1945, and occurs in sugarcane growing regions worldwide [1, 2]. RSD infection generally causes 5–30% yield loss, but it can go up to 60% in severely affected ratoon crops [3, 4].

Sugarcane is the most important sugar crop in the world, accounting for about 80% of sugar production globally, and over 90% in China [5, 6]. China is the fourth largest sugar producer in the world. RSD was first reported in China in 1986 [7]. The incidence of RSD in commercial sugarcane crops in China ranges from about 65 to close to 90%, depending on the region [6]. However, it could be significantly higher with recurring and increasingly severe drought.

RSD is a bacterial disease caused by the gram-positive bacteria *Leifsonia xyli* subsp. *xyli* (*Lxx*) [8]. There are no easily distinguishable visual lesions or similar symptoms for RSD. A general growth reduction, decreased stalk diameter and shorter internode length is a common feature of RSD. Because water stress accelerates RSD pathogenesis, the RSD-induced growth reduction is often discounted as drought and/or nutritional deficiency responses, leaving the disease undetected and unchecked. *Leifsonia xyli* subsp. *xyli* is a slow growing and nutritionally fastidious bacterium making it difficult to culture. It takes nearly a month to grow it into a visible colony on solid medium [9, 10]. The predicted *Lxx* pathogenicity genes include those for cellulase, pectinase, wilt-inducing protein, lysozyme, and desaturase, of which the latter is involved in the synthesis of abscisic acid, a hormone that arrests growth [11]. *Lxx* infects its host mainly through wounds on the stalk and is highly infectious. For instance, a previous study showed that diluting the bacteria-containing sap by several hundred-fold still retains strong infectivity [12]. Because *Lxx* mainly inhabits the xylem vessels of sugarcane, RSD has been long recognized as a disease limited to the xylem [13]. Therefore, most of the studies on RSD focused on stem [14–16]. Recent studies using a GFP-labeled *Lxx* strain to track *Lxx* colonization in the whole sugarcane plant found its presence in leaf mesophyll cells as well as those surrounding the vascular bundle sheath [17].

Currently, RSD is mainly controlled by pathogen-free planting material, maintaining good farm hygiene through appropriate field management and strict implementation of quarantine practices [18]. However, some control methods, such as meristem tissue culture are time-consuming and resource-intense, but becoming increasingly popular. Previous studies have shown genotypic variation for resistance to RSD [19]. Nonetheless, breeding for RSD is not a priority in most breeding programs. Hot water treatment, where seedcanes are soaked in hot water maintained at 50°C for two hours is a common method to reduce *Lxx* titre. This method, while economical and practiced widely for commercial cropping, does not completely eliminate *Lxx* [20].

Previous studies have reported reduced water potential and increased membrane permeability and free amino acid levels in sugarcane following *Lxx* infection [21]. *Lxx* infection also affects enzymes involved in plant defenses such as superoxide dismutase (SOD), peroxidase (POD) and catalase (CAT) [16, 22], and alters the level of endogenous plant hormones indole-3-acetic acid (IAA), gibberellic acid (GA) and abscisic acid (ABA) [14, 23]. Further, photosynthesis was found decreased in *Lxx*-infected plants [22, 24]. We observed changes in leaf and stalk anatomy as well as morphological and structural variation in chloroplasts of *Lxx*-infected sugarcane [15, 23]. The genetic elements and mechanisms causing structural and physiological responses of sugarcane to *Lxx* infection are not well understood. Considering the extent of growth and developmental inhibition occurring in *Lxx*-infected sugarcane, it is highly likely that a number of growth- and pathogenicity-related gene networks may be involved in eliciting disease symptoms. Studying global gene expression is an effective and useful approach in understanding the molecular basis of pathogenesis, and deep transcriptome sequencing technology, RNA-Seq, has been used to investigate disease resistance mechanisms in various plant species [25, 26]. RNA-Seq analysis of rice transcriptome 24 h post-inoculation with blast fungus found up-regulation of 240 genes encoding secreted proteins, including glycosyl, hydrolases and cutinases [27]. RNA-Seq has also been widely used investigate sugarcane abiotic stresses [28, 29], developmental processes such as sugar accumulation [5, 30], self-defoliation [31] and diseases [32–35].

To date studies on RSD have mostly concentrated on developing disease prevention methods [36], diagnostics [37], pathogenesis-related host physiology [14–16, 21–24] and pathogen genome analysis [38]. There is rarely report on global gene expression analysis of healthy and *Lxx*-infected sugarcane. Also, despite sequencing *Lxx* genome, and considerable *Lxx* mutagenesis studies, little is known about the genetic architecture of *Lxx*-sugarcane pathological interactions [11]. RSD pathogenesis-related mechanistic studies are further disadvantaged by the lack of clear and consistent visual disease symptoms other than a generalized growth inhibition of infected plants. With the recent advancements in molecular tools and technology it is now possible to gain insights into molecular aspects of host-pathogen interactions at gene level [33, 39]. The main objective of this study is to identify RSD-associated gene networks and possible reasons for *Lxx*-induced growth inhibition. Also, it is expected that, as with other diseases, comprehensive gene expression analyses will start decoding the RSD host-pathogen interaction black-box and pave the way for future research on molecular targets, including molecular markers, for genetic improvement of sugarcane for RSD resistance. For this study, we used a *Lxx*-susceptible sugarcane variety Badila and conducted RNA-Seq analyses to identify genetic elements and networks associated with RSD pathogenesis in sugarcane.

## Materials and methods

### Materials and inoculation

Healthy, disease-free seedcanes of sugarcane cultivar Badila (*Saccharum officinarum* L.), which is highly susceptible to RSD, were used for this study. Seedcanes were grown in the greenhouse at the Agricultural College, Guangxi University, Nanning, China. RSD-free status of experimental materials was confirmed by *Lxx* diagnostic PCR assay. The stalk of seedcane plants were cut into single-bud nodal cuttings, called seedcane setts, with sterile knife and immersed in hot water maintained at 50°C for 2 h to further ensure that they are *Lxx*-free. The *Lxx* strain used for inoculation was *Leifsonia xyli* subsp. *xyli* GXBZ01 (Accession number LFYU00000000). Cultures of this bacterium were prepared as described previously [23]. PCR assay to detect *Lxx* was performed as described elsewhere [21]. The two cut surfaces of seedcane setts were inoculated with 300 μL *Lxx* solution (10^8^ cfu/mL). The control group was inoculated with MSC liquid medium. The seedcane setts were kept at 28°C for two days and then planted in sandy soil. *Lxx*-inoculated and control seedlings at the third-leaf-stage were tested for RSD-infection by PCR assay. The control (*Lxx*-free) and infected plants were then transferred to pots (height × diameter, 350 mm × 300 mm) containing 18 kg of composite soil (soil: sand: organic fertilizer, 6:2:2). Six pots, each carrying three plants, were maintained for each treatment group. At 90 days post-inoculation, top visible dewlap leaf (designated as leaf +1) and basal part of stalk were sampled from three plants selected randomly from the infected and control groups. Collectively there were four groups of samples, each with three biological replicates, totaling 12 biological samples. The four groups of samples were named healthy leaves (HL), healthy stalks (HS), *Lxx*-infected leaves (IL) and *Lxx*-infected stalks (IS), respectively. All the samples were frozen in liquid nitrogen immediately after collection and then stored at -80°C until used for analyses.

### RNA-seq library construction

Healthy and *Lxx*-infected stalks and leaves collected on day 90 post-inoculation were used for high-throughput transcriptome sequencing. Total RNA was extracted using the TRIzol^®^ method [40]. The integrity of the extracted RNA was confirmed using 1% agarose gel electrophoresis. A was used to test the Purity of the extracted RNA was determined by spectrophotometry, using NanoDrop 2000 (Thermo Scientific, Waltham, USA). Fifteen μg of good quality RNA from each sample was used for cDNA library construction. The quality of the cDNA library was determined using an Agilent 2100 Bioanalyzer and ABI StepOnePlus Real-Time PCR system. The cDNA libraries that passed quality assessment were used for sequencing. The cDNA library construction and high-throughput sequencing were conducted at BGI-Shenzhen. An Illumina HiSeq 2000 platform was used for sequencing, employing three biological replicates for every type of tissue used in the experiment.

### De novo transcriptome assembly

As the first step, the raw reads were filtered to remove low-quality reads, adaptors, and the reads with high Ns to generate clean reads. From clean reads de novo transcripts were assembled (PCR redundancy was removed to improve the assembly efficiency) using Trinity software [41]. The assembled transcripts were further processed using Tgicl for clustering and redundancy removal and unigenes were generated.

### Gene annotation

Unigenes obtained from the assembly were functionally annotated using seven databases; they are KEGG (Kyoto encyclopedia of genes and genomes), GO (Gene ontology), NR (NCBI non-redundant database), NT, Swiss-Prot, Pfam, and KOG (EuKaryotic Orthologous Group) [42].

### Analyses of gene expression levels and Differentially Expressed Genes (DEGs)

The clean reads were mapped back to the assembled sequences using Bowtie2 [43], followed by analysis of gene expression level in each sample using RSEM (RNA-Seq by Expectation-Maximization) [44]. Fragments per kilobase of transcript per million mapped reads (FPKM) were used to normalize the gene expression level of every transcript in each sample. The DEGs in sugarcane after *Lxx* infection were identified using edgeR software [45]. The differentially expressed transcripts were selected by applying stringent parameters: i) FPKM > 1, ii) Log_2_FC (log_2_ fold change) > 1 (up-regulated) or Log_2_FC < -1 (down-regulated), iii) p-value < 0.05 and iv) FDR (false discovery rate) < 0.05.

### GO and KEGG pathway enrichment analysis

Based on the results of GO and KEGG annotation, as well as official classification, the DEGs were classified (FPKM > = 10: at least one of the three biological replicates of each group of samples had an FPKM > = 10) into groups of cellular function and biological pathway. Meanwhile, R’s phyper function was used for GO and KEGG enrichment analysis. The p-values were calculated and subjected to FDR calibration and the functions or pathways showing significant enrichment were identified using a FDR ≤ 0.01.

### Real-time quantitative RT-PCR (RT-qPCR)

Reverse transcription quantitative real-time polymerase chain reaction (RT-qPCR) was performed to quantify the expression levels of the transcripts obtained from high-throughput sequencing. First, TRIzol^®^ (Cowin Biosciences) was used to extract total RNA, followed by RNA quality assessment using NanoDrop 2000. cDNA reverse transcription was performed using a TAKARA PrimeScriptTM RT reagent kit (TaKaRa). RT-qPCR was performed using SYBR Premix Ex Tap^TM^ II (TaKaRa) using LightCycler^®^480 II (Roche Applied Science). The PCR reaction mixture and thermal profile were set up as described by Zhu et al. [21]. Gene relative expression levels were calculated using the 2^−ΔΔCt^ method [46]. Primers of candidate genes and an internal reference gene (GAPDH, Glyceraldehyde-3-phosphate dehydrogenase) were designed using Primer 5.0 software and synthesized at Sangon Biotech (Shanghai) Co., Ltd. The primer sequences used are listed in S1 File.

## Results

### Transcriptome assembly

To study the response of sugarcane to *Lxx* infection at gene level, we performed RNA-Seq of leaf and stalk tissues of *Lxx*-infected and non-infected plants using an Illumina HiSeq 2000 platform with pair-end sequencing technology. After data filtering, 79.73 Gb high-quality clean reads were obtained. De novo assembly of clean reads using Trinity software produced 107,750 unigenes after redundancy removal. The total assembled unigenes contain 165,562,488 nucleotides (approximately 165 Mb), with an average length of 1,536 bp, an N50 value of 2,200 bp, and GC content of 50.73% (Table 1). Within the 107,750 unigenes, 64,135 unigenes (59.52%) were longer than 1,000 bp, among which 11,720 unigenes (10.87%) were longer than 3,000 bp. The distribution of unigenes length is shown in S1 Fig.

**Table 1:** Overview of the assembled sugarcane transcriptome from healthy and *Leifsonia xyli* subsp.*xyli* infected plant tissues.

All-Unigene type	Result
Clean reads (Gb)	79.73
Total Number	107,750
Total Length (bp)	165,562,488
Mean length	1,536
N50 length	2,200
N70 length	1,611
N90 length	839
GC (%)	50.73
TransDecoder identified CDSs	72,733

Benchmarking Universal Single-Copy Orthologs (BUSCO, https://gitlab.com/ezlab/busco) was used to analyze the assembly results of each sample from 12 groups. It was found that the completeness of all assemblies was > 89%; samples HS-1, HS-2, HS-3, and IS-1 were the best, with > 96% completeness. After removing redundant reads, all samples had a completeness of 97%. Among these, 290 genes were complete, 4 were partially recovered (fragmented) and only 9 were missing (S2 Fig). These results also showed that the de novo assembly of transcripts from the 12 samples covered the majority of sugarcane genes, and the assembly results were suitable for further analyses and experiments.

### Transcriptome annotation and analysis of Differentially Expressed Genes (DEGs)

**Fig 1:**
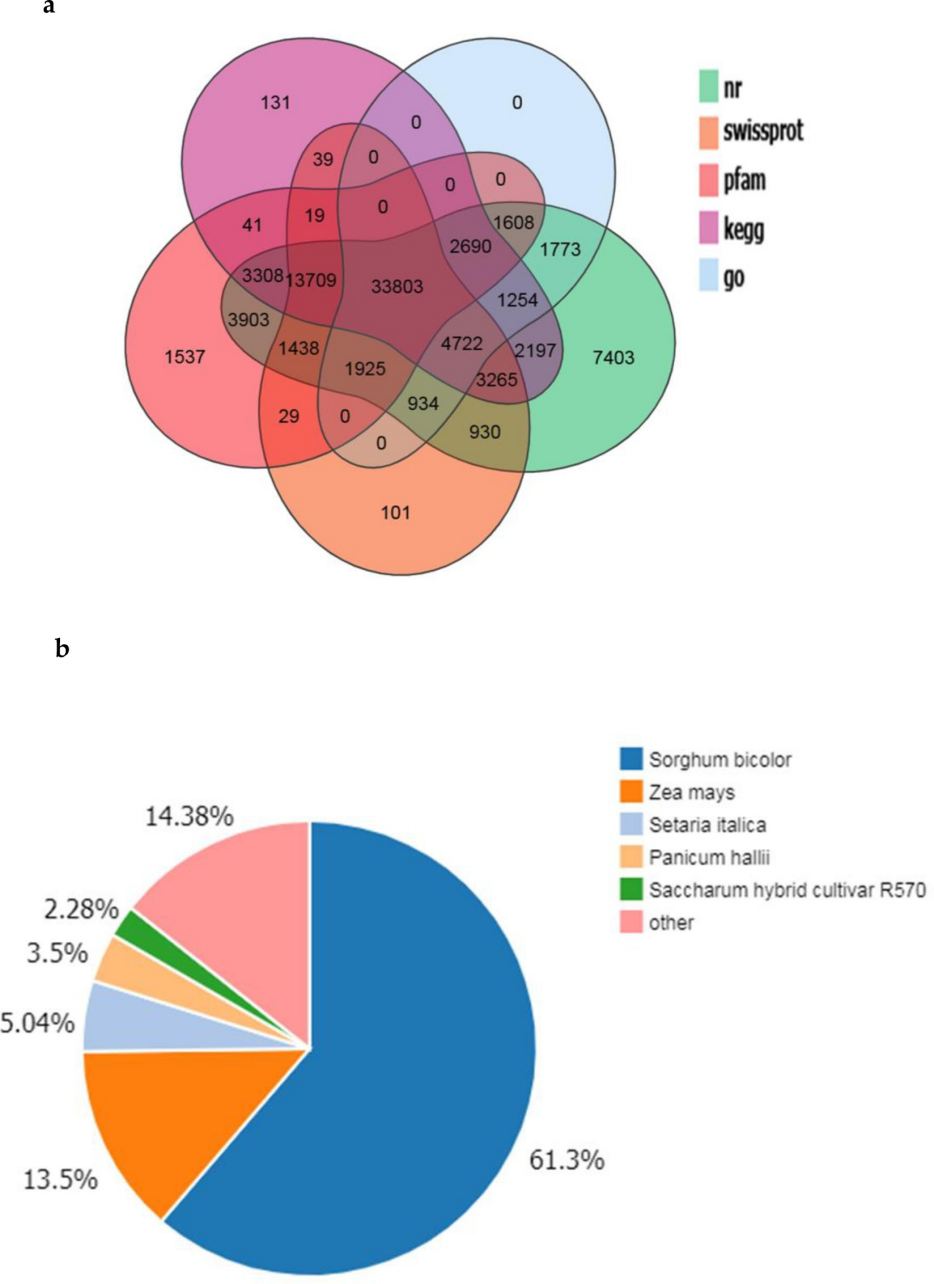
Annotation of the assembled sugarcane transcriptome. (a) Venn diagram of genes and predicted proteins aligned to different databases; (b) Distribution of species aligned by the assembled sugarcane genes.

Multiple databases were used to perform functional annotations of the transcripts obtained from the assembly. Among all the databases used, 84,862 transcripts (78.76% of all transcripts) were matched to NR, which showed the largest number of matched sequences, followed by KEGG (65,178, 60.49%). In addition, 464,010 (59.41%), 60,914 (56.53%), and 8,709 (45.21%) transcripts were matched to Pfam, Swiss-Prot, and the GO database, respectively (Fig 1A). Further analysis of NR annotations showed that 52,010 (61.3%) were matched to *Sorghum bicolor*, 11,459 to *Zea mays* (13.5%) and 4,273 to *Setaria italica* (5.04%) (Fig 1B). Of these three species, *S*. *bicolor* and *Z*. *mays* are closely related to sugarcane, which further attests the quality and reliability of the assembly.

**Fig 2:**
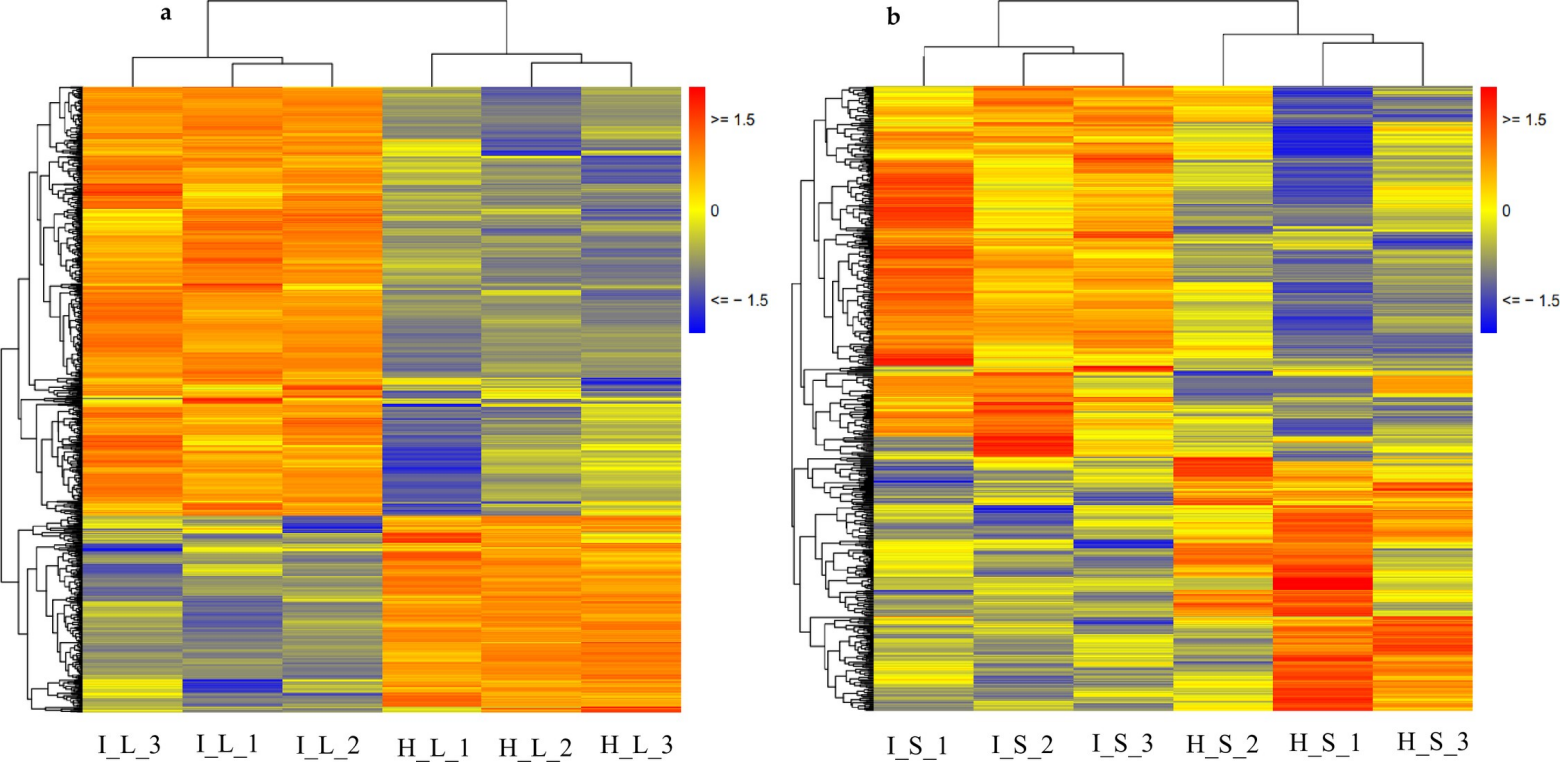
Heat map of all transcripts of (FPKM>1) (a) sugarcane leaves; (b) sugarcane stalk.

In the four sample groups, namely, HL, IL, HS, and IS, approximately 77,204 transcripts showed an FPKM≥1, and at least 20% of transcripts in the corresponding sample groups had FPKM≥10 (Fig 2). The distribution of FPKM values was shown in S3 Fig. Pearson correlations for the replicates were above 0.95 in HL, HS, IL, and IS samples (S4 Fig), indicating the reproducibility of replicates. DeSeq software (http://www.bioconductor.org/packages/release/bioc/html/DESeq.html) was used to analyze the differential expression of transcripts among the experimental groups. A large number of DEGs was identified in the *Lxx*-infected leaves (11,802; 7,721 up-regulated and 4,081 down-regulated) and stalks (9,325; 4,266 up-regulated and 5,059 down-regulated), compared to the corresponding tissues from healthy plants. In the leaves, about two-third of the DEGs were up-regulated while in the stalk more DEGs were down-regulated. To identify important DEGs among different groups, we applied filters to obtain DEGs with |Log_2_FC|≥1, and an average FPKM value≥10.

### Functional analysis

**Fig 3:**
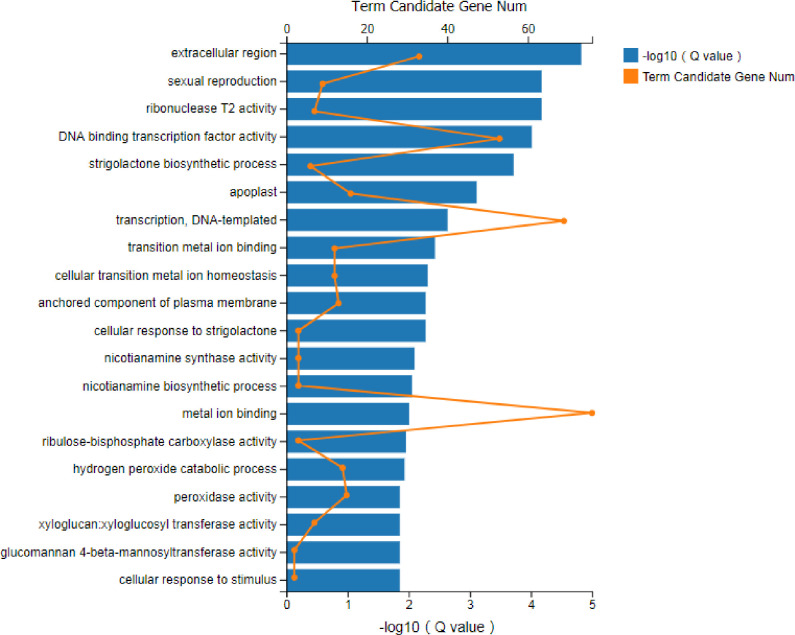
GO enrichment analysis of DEGs (FPKM> = 10) in *Lxx*-infected and healthy sugarcane. The DEGs functions are displayed with respect to their statistical significance (Q value < 0.05). “Term candidate gene num” means the number of genes enriched in each GO term category.

**Fig 4:**
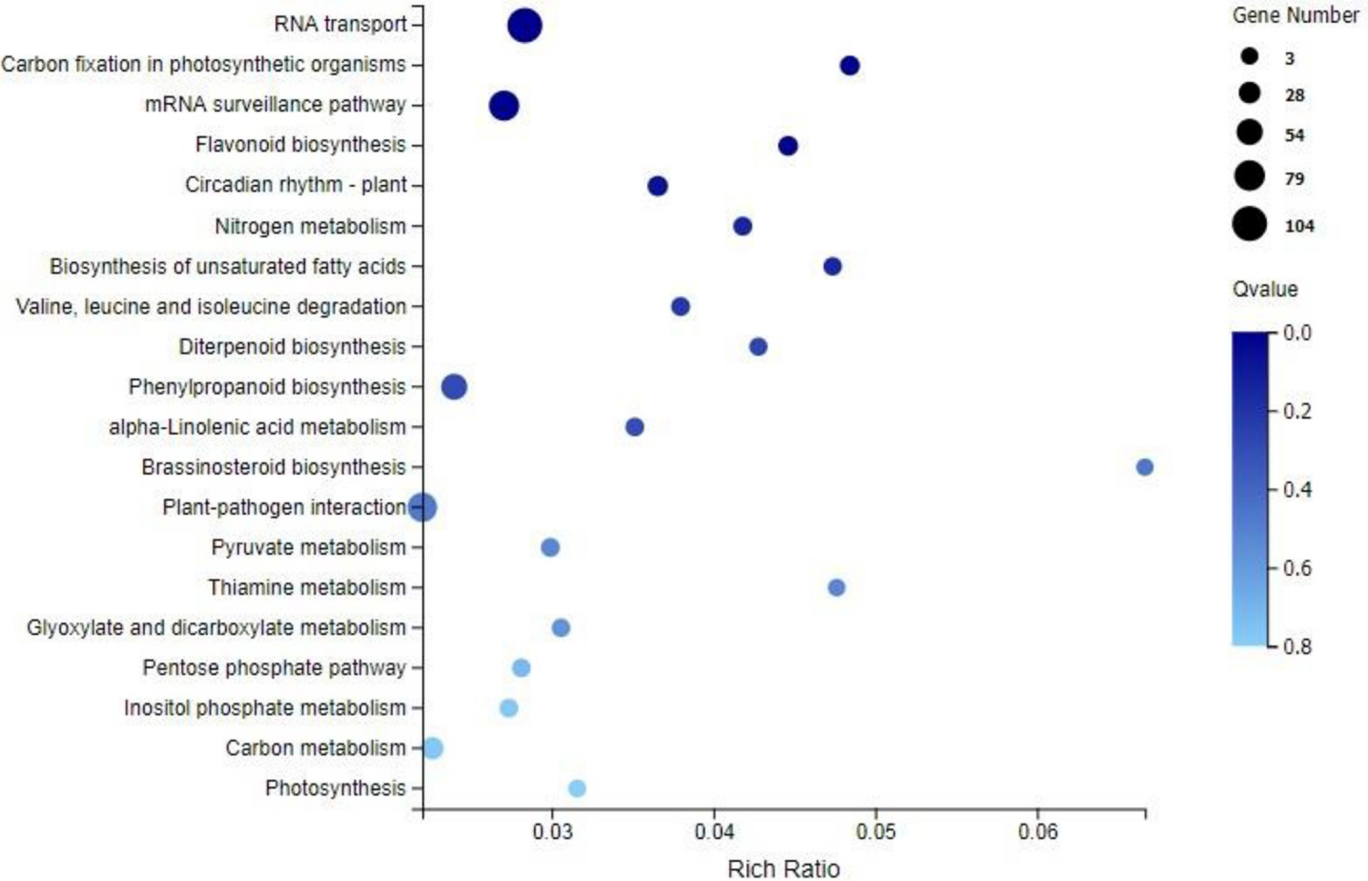
KEGG enrichment analysis of the DEGs (FPKM> = 10) in *Lxx*-infected and healthy sugarcane. Rich Ratio, the ratio of the number of genes annotated to an entry in the selected gene set to the total number of genes annotated to the entry in this species, Rich Ratio = Candidate Gene Num / Gene Num; Bubble size represents the number of genes that are annotated on a KEGG Pathway, color represents enrichment Qvalue, and darker color represents smaller Qvalue lighter color represents higher Qvalue.

Functional analysis was performed to identify the enriched GO terms and KEGG pathways associated with DEGs (FPKM>10) in sugarcane affected by RSD. Among the significant GO terms (Fig 3), 69 genes were involved in “metal ion binding” (GO: 0046872), 12 genes were related to “DNA binding transcription factor activity” (GO: 0003700), and 15 genes were enriched for “peroxidase activity” (GO: 0004601). Next, a bubble plot was used to identify the significant KEGG pathways related to DGEs (Fig 4), and the result showed that 104 DEGs were involved in “RNA transport” (ko03013), which was the most significant one. Interestingly, 23 DEGs were found involved in “photosynthesis” (ko00195) and “carbon fixation in photosynthetic organisms” (ko00710). Besides, 73 DEGs were found to be involved in the pathways linked to “plant-pathogen interaction” (ko04626). In addition, plant hormone-related pathways such as “flavonoid biosynthesis” (ko00941; 17 DEGs), “diterpenoid biosynthesis” (ko00904; 8 DEGs), “phenylpropanoid biosynthesis” (ko00940; 53 DEGs) and “brassinosteroid biosynthesis” (ko00905; 3 DEGs) were also found to be involved in *Lxx* infection.

### Sugarcane genes in response to *Lxx*

**Fig 5:**
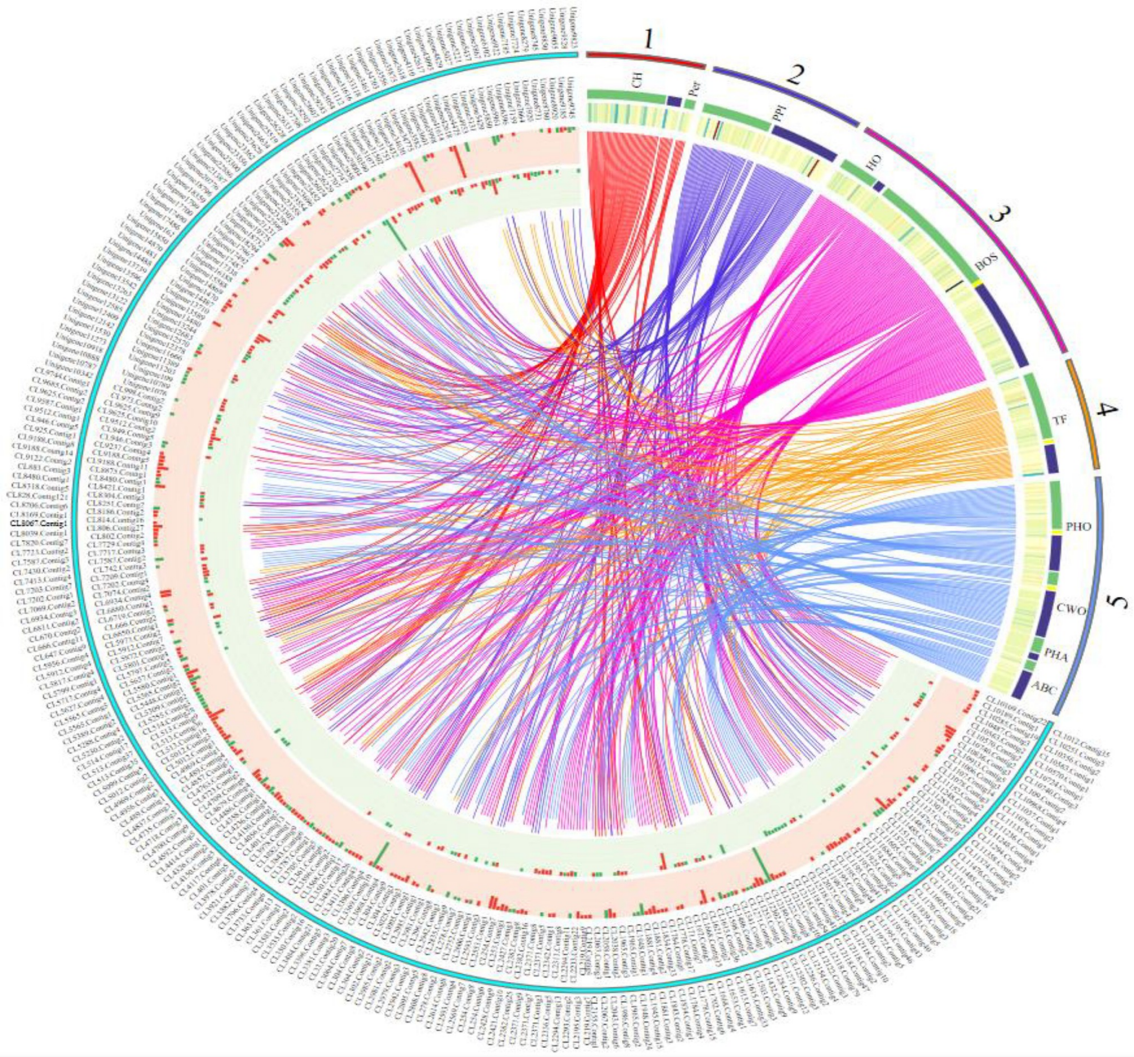
Circular map of crucial genes in response to *Leifsonia xyli* subsp. *xyli*. The inside and outside of the cyan coils represent the gene IDs of DEGs; the corresponding middle circle and the innermost circle represent the gene expression in leaves and stems respectively; the red one represents the up-regulation, and the green one represents the down-regulation. The numbers 1, 2, 3, 4, 5 represent photosynthesis, plant disease interactions, hormone and secondary metabolism, transcription factors, and other genes (genes related phosphorylated, cell walls and ABC transports), respectively. The corresponding green and blue colors represent leaves and stems, respectively.

The DEGs (FPKM>10) in different functional categories that might be related to sugarcane in response to *Lxx*, such as photosynthesis, phytohormone biosynthesis and metabolism, secondary metabolism related proteins, transcription factors, pathogenesis-related (PR) proteins and some other proteins were analyzed, and the data of these gene families are shown in Fig 5, including the number of genes identified and those involved in various metabolic pathways.

#### Photosynthesis-related genes

It was found that, with *Lxx* infection, the activity of 51 photosynthesis-related DEGs in leaves (33 up-regulated, 18 down-regulated) and 8 in stalks (5 up-regulated, 3 down-regulated) were altered. However, there were no common photosynthesis-related DEGs present in both leaves and stalks (S2 File; Fig 5). In Ko00195, which is a photosynthesis-related pathway, the genes encoding PSI (*PsaB*, unigene8780), PSII (*PsbH*, unigene1470), F-type ATPase (alpha subunit, CL2043.Contig6), and cytochrome b6-f complex (*PetN*, unigene23696) were all up-regulated, whereas the genes encoding photosynthetic electron transport proteins (*PetH*, CL946.Contig3, and CL946.Contig5) were down-regulated in the leaves. In another pathway related to photosynthesis, Ko00196, the genes encoding antenna proteins (e.g., CL1486.Contig3) were also down-regulated in the leaves. However, these DEGs were not detected in stalks (Fig 5).

Further analysis showed that, out of the 51 DEGs from leaves, 45 were related to chloroplasts (Fig 5). Three chlorophyll-related DEGs were detected in the *Lxx*-infected leaves. Among them, CL1486.Contig3, which was annotated as a light-harvesting protein complex, exhibited down-regulated gene expression. The other two genes, CL5309.Contig2 and unigene8780, however, up-regulated. Following *Lxx* infection, the expression of *RuBP* (Ribulose bisphosphate), a key enzyme in photosynthesis, was down-regulated in the leaves.

#### Signaling pathways in plant-pathogen interactions

In *Lxx*-infected plants, 5% of the total DEGs were found to be involved in the plant-pathogen interaction pathway (KO04626) with 35 and 38 DEGs expressing in the leaves and stalks (S2 File; Fig 5), respectively. No common DEGs between leaves and stalks were detected. Overall, most of the plant-pathogen interactions DEGs encode calcium-dependent protein kinases (CDPKs), pathogenesis-related (PR) proteins, and defense-related TFs. It was found that 48 CDPK-related genes were differentially expressed, with 23 DEGs in the leaves and 25 in the stalks, which comprise 65.75% of DEGs in the plant-pathogen interaction pathway (Ko04626) (S2 File; Fig 5). PR proteins are closely linked to the induced resistance in plants. Seven DEGs identified were found to be PR proteins in response to *Lxx* infection. Interestingly, most of these DEGs (6) were found in the stalks and down-regulated. In addition, two genes of pathogen-associated molecular patterns (PAMPs) were found in the leaves. It was also found that some *ZFP* (Zinc Finger Protein) and *NBS-LRR* (Nucleotide binding site-leucine-rich repeats) genes, which had been reported as disease resistance genes, were up-regulated in the *Lxx*-infected sugarcane. Besides, heat shock proteins and transcription factors were also found to be involved in the sugarcane-*Lxx* interactions.

#### Genes related to phytohormone and secondary metabolism

It was found that as many as 167 DEGs in *Lxx*-infected plants were related to phytohormone and secondary metabolism (S2 File). Among these DEGs, 102 genes were from the leaves, 68 from the stalks, and 3 were present in both. In this analysis, 38 DEGs, including 27 in the leaves and 12 in the stalks, are involved in the phytohormone signal transduction pathway (Ko04075). The CL1615.Contig7 gene is particularly interesting because it showed differential expression in both leaves and stalks, but with opposite trends. Its expression was up-regulated 1.27-fold in the leaves and down-regulated 1.05-fold in the stalks (Fig 5). Functional annotation of CL1615.Contig7 indicated that it is a gene encoding SRK2, a Ser/Thr receptor kinase involved in plant development and defense. The DEGs in the phytohormone signal transduction and metabolism pathway (Ko04075) are mainly involved in the biosynthesis of auxin, gibberellin (GA), ethylene (ET), abscisic acid (ABA) and jasmonic acid (JA) (S2 File). Eighteen percent of all the phytohormone-related DEGs identified in response to *Lxx* infection were gibberellin-related and 6 of them were in the leaves and 1 in the stalks (S2 File; Fig 5). The negative regulator of GA signaling, *DELLA* (CL5565.Contig1, CL5565.Contig2, and CL11925.Contig5) [47] was up-regulated, whereas *GID1* (GA-insensitive dwarf 1) (CL1271.Contig6 and CL1271.Contig12) [48] that encodes GA receptors, was down-regulated (Fig 5). Of the other phytohormone-related DEGs, 6 genes were found to encode auxin-related proteins, including auxin responsive factors (ARFs), indoleacetic acid-related proteins (IAAs), and auxin-induced proteins, of which 5 were up-regulated and 1 down-regulated. Among the other phytohormone-related DEGs, 5 genes were related to ET, and all were up-regulated. PYR/PYL, the negative regulators in upstream ABA signal transduction [49], was found up-regulated during *Lxx* infection in sugarcane. It was also found that there were 5 DEGs that were related to JA biosynthesis, among which only one, in the stalk, was up-regulated. In leaves, except for the CL3369.Contig10 gene, which was down-regulated, all the DEGs were up-regulated, including *MYC* (CL9122.Contig2), a central regulator in the JA responsive pathway [50].

In *Lxx*-infected sugarcane, 16 flavonoid-related DEGs were found in the leaves, and two in the stalks; also, 5 phenylalanine-related DEGs were observed in the leaves and one in the stalks (Fig 5). In leaves, the expression of these DEGs was mainly up-regulated. The expressions of *PAL* (CL514.Contig17, CL514.Contig28), *4CL* (CL4056.Contig2, CL12037.Contig4), *CHS* (CL361.Contig1, CL361.Contig6), and *CHI* (CL10487.Contig3, CL11236.Contig1) were all up-regulated in leaves.

#### Regulation by transcription factors

It was found that the DEGs related to TFs were mainly AP2/EREBP, MYB, WRKY, and MAPK transcription factors (S2 File). In addition, the MADS-box and NAC transcription factors in sugarcane were also differentially expressed in *Lxx*-infected plants. In *Lxx* infected plants 23 AP2/EREBP transcription factor genes were differentially expressed, comprising 37.1% of all transcription factor DEGs found in this study. There were 14 AP2/EREBP transcription factor-encoding DEGs in the leaves, 11 in the stalks, and 2 in the both (unigene2858_All and unigene17492_All). Besides, differential expression of 7 MYBs and 5 WRKY genes were found associated with *Lxx*-infection in sugarcane (S2 File). Analysis showed that upon *Lxx* infection, 6 *MAPK* and 3 *MADS-box* genes were differentially expressed in the leaves and only 1 each of *MAPK* and *MADS-box* genes in the stalks. The *NAC1* (unigene7159_All) was up-regulated in stalks upon *Lxx* infection.

#### Other genes involved in *Lxx* stress responses

A total of 36 protein phosphorylation/dephosphorylation genes in sugarcane were differentially expressed in response to *Lxx* infection (S2 File; Fig 5). It was also found that the genes related to phagosomes and lysosomes had altered expression levels (Fig 5). Following *Lxx* infection, many genes involved in cell wall strengthening were upregulated (Fig 5), including 6 cellulose synthase (*CesA*) genes, all of which were found in stalks. In addition, 9 glycosyltransferases (*GTs*) in stalks and 1 GT in the leaves were up-regulated, as well as pectin acetylesterase and polygalacturonate 4-alpha-galacturonosyltransferase (*GAUT1*), both of them are related to pectin biosynthesis. Pectinesterase (*PME*) genes, which are related to pectin degradation, were down-regulated.

### RT-qPCR

**Fig 6:**
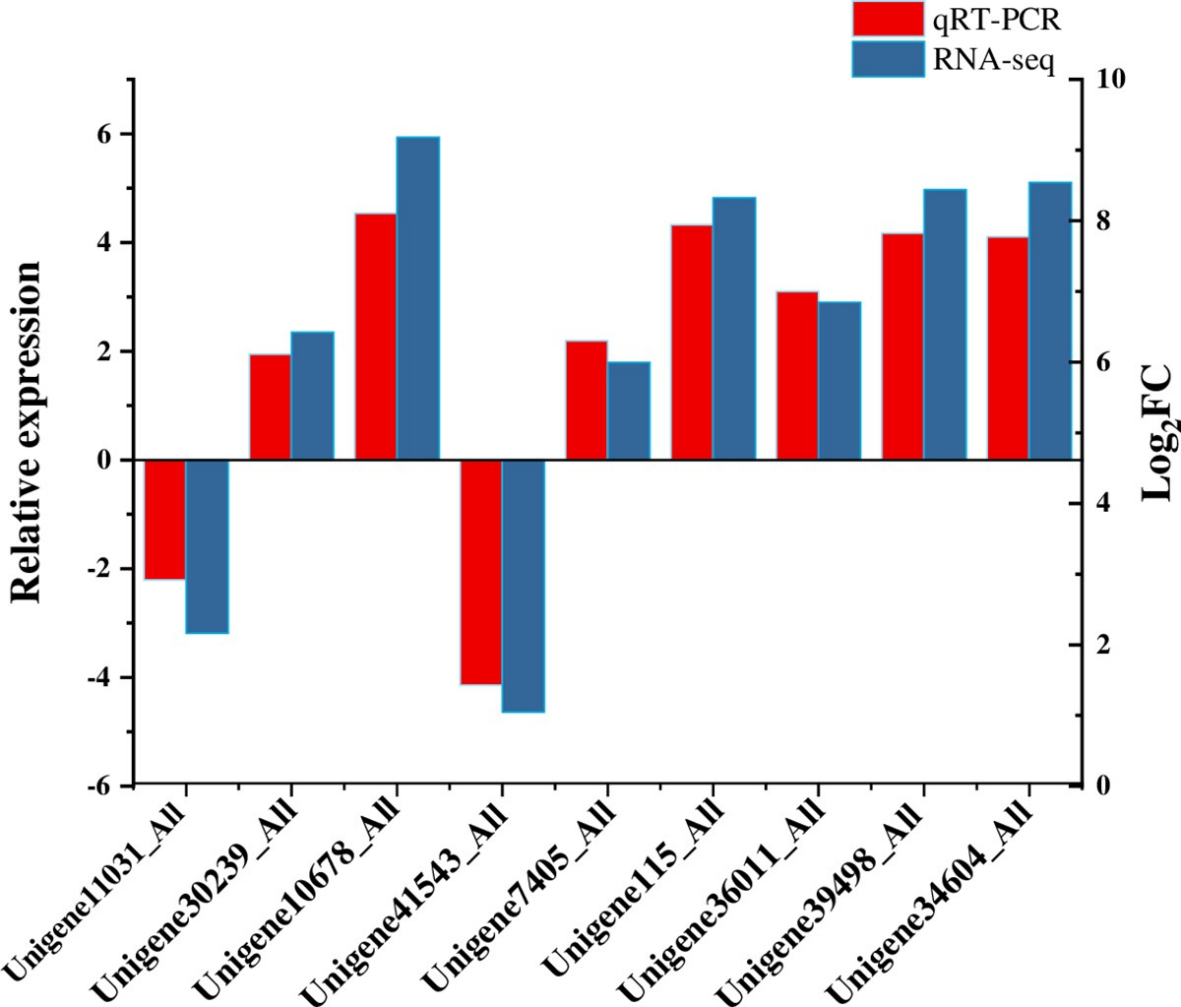
qRT-PCR validation for 9 candidate transcripts. Error bar represents the standard deviation.

RT-qPCR was used to validate gene expression in healthy and *Lxx*-infected sugarcane in this study. Nine transcripts were randomly selected for RT-qPCR with three technical replicates. These genes are specifically involved in photosynthesis (Unigene11031_All, Unigene30239_All), plant and pathogen interaction (Unigene10678_All, Unigene41543_All), phytohormones (Unigene7405_All, Unigene115_All), cell walls (Unigene36011_All, Unigene39498_All), and secondary metabolism (Unigene34604_All). The qRT-PCR results (Fig 6) showed similar expression patterns compared to RNA-Seq quantification results. Overall, the up-down expression trend of DEGs was basically consistent both in qRT-PCR and RNA-seq results, indicating the qRT-PCR results support RNA-Seq quantification results.

## Discussion

In this study, paired-end sequencing technologies were used to investigate the molecular changes occurring at gene activity level in sugarcane leaves and stalks in response to *Lxx* infection. This is the first transcriptome study on different RSD-infected sugarcane organs using the deep sequencing technology. Analysis of DEGs (FPKM>10) between HL and IL and between HS and IS has identified 839 DEGs between HL and IL and 610 DEGs between HS and IS with only 21 being common across both comparisons. Stalks and leaves are structurally and functionally very different organs and not surprisingly their gene expression profiles, as shown in this study, are considerably different.

It was found that many DEGs in infected plants are related to sugarcane photosynthesis. Most of the photosynthetic reactions take place in chloroplast, and the number of chloroplast-related DEGs discovered in the diseased plants indicates that *Lxx* infection significantly affected chloroplasts. The majority of these chloroplast-related DEGs were chloroplast membrane genes and *RuBPcase*. The chloroplasts in RSD-infected leaves were deformed, most chloroplasts grana were disappeared, and outer and inner membrane of chloroplasts were deformed [15]. These results [15, 23] are consistent with the down-regulation of chloroplast gene transcription observed in this study. Collectively, these findings suggest that *Lxx* infection severely disrupts chloroplast function, which in turn affects plant growth. Under pathogen stress, antioxidant substances could help maintain the production and availability of adenosine triphosphate (ATP) and nicotinamide adenine dinucleotide phosphate (NADPH) by preserving photosystem function to fix carbon [51]. Upon *Lxx* infection, the genes encoding POD, SOD and CAT were up-regulated, indicating plant’s defense to maintain photosynthesis to some extent.

During plant–pathogen interactions, plants trigger a series of defense mechanisms to protect themselves, which include changes in antioxidant enzymes, increase in PR proteins, and phytoalexin biosynthesis [52]. Calcium-dependent Protein Kinases (CDPKs), involved in the regulation of a myriad of growth and developmental process, signal transduction and pathogenesis, exist widely in eukaryotic cells [53, 54]. In this study, the expression of CDPKs was altered considerably, indicating that CDPKs play critical roles in the signaling transduction of defense responses to *Lxx* infection in sugarcane. This was evident from the increased expression of some of the disease resistance genes such as *ZFP* and *NBS-LRR* observed in the present study.

The role of plant hormones salicylic acid (SA), JA, and ET in plant defense responses to pathogens has been extensively studied. Ethylene is involved in regulating plant growth and development, as well as plant responses to biotic and abiotic stresses. Abscisic acid also plays a role in plant-pathogen interactions, mostly as a negative regulator of plant disease-resistance [55, 56]. In this study *PYR* and *PYL* genes, the negative regulators acting upstream in ABA signal transduction [49], were found up-regulated during *Lxx* infection. This is possibly a component of defense strategy operating in sugarcane in response to *Lxx* infection. However, generalized stunting of plant growth is the most prominent visual symptom of RSD and GA is a major regulator of sugarcane growth and development [57]. In *Lxx*-infected sugarcane, up-regulation of *DELLA*, the negative regulator GA response, and down-regulation of *GID1*, which facilitates DELLA protein degradation, were observed. *Lxx* infection-induced inhibition of GA action thus may be a key growth regulatory mechanism in RSD affected plants as observed in other crops infected with different pathogens [58, 59].

Plant secondary metabolites play roles in transducing environmental and biotic stress signals. Many DEGs detected here are involved in the biosynthesis of secondary metabolites, mainly flavonoids and phenylalanine biosynthesis. Phenylalanine is converted into trans-cinnamic acid, catalyzed by L-phenylalanine ammonia-lyase (PAL), which is then converted into 4-hydroxycinnamic acid by cinnamic acid-4-hydroxylase (C4H) and then to 4-coumaroyl-CoA catalyzed by 4-coumaroyl-CoA ligase (4CL), which is used to synthesize alkaloids, lignin, and flavonoids by chalcone synthase (CHS) and chalcone isomerase (CHI) [60]. In this study, the expression of the *PAL*, *4CL*, *CHS*, and *CHI* genes were upregulated during *Lxx* infection, suggesting that sugarcane responded to *Lxx* infection by increasing the production of polyphenols and other related secondary metabolites with antibacterial properties.

In response to pathogen infection, plants activate numerous transcription factors that are associated with various plant defense strategies such as induced cell death, growth reduction to conserve energy, anatomical modifications and production of antimicrobial agents [61]. Among the major plant transcription factors, APETALA2/ethylene-responsive element binding proteins (AP2/EREBP) form an ancient transcription factor superfamily, of which the AP2 transcription factors regulate reproductive development, while EREBP transcription factors elicit ET and ABA and stress responses [62, 63]. MYB transcription factors are involved in SAR and HR during defense responses [64] while transcription factors WRKY, MAPKs, NAC and MYB were implicated in both biotic and abiotic stress responses and regulation of growth and development [64–69]. In our study, AP2/EREBP, NAC, WRKY, MYB and MAPK showed remarkable differential expression in *Lxx*-infected plants, suggesting the activation of gene networks controlling pathogenesis, disease resistance and growth and development, which tallies well with the growth variation following *Lxx* infection.

Protein phosphorylation serves as a molecular switch in cellular signal transduction and the regulation of enzyme activity [70]. In this study, many DEGs are involved in protein phosphorylation, indicating the large variation in protein phosphorylation/dephosphorylation occurring in *Lxx*-infected sugarcane. For instance, DEGs related to phagosomes and lysosomes were detected, which suggests that sugarcane cells may be defending the infection of *Lxx* through endocytosis. It is well-known that plant cell walls serve as the first physical barrier to pathogen infection. A previous report has characterized the genomic islands in *Lxx* genome that produce pectinase and cellulase involved in cell wall degradation [38] to initiate infection and to channel nutrients from the host. In *Lxx*-infected sugarcane, pectinases and cellulases first degrade the middle lamella and then gradually disintegrate the cell wall. The degraded cell walls with partial deformation produced large amounts of debris that block the pit membrane in host xylem vessels, causing xylem occlusion commonly noticed in RSD-affected sugarcane. This blockage reduces the ability of sugarcane xylem vessels to transport nutrients and water, which exacerbate plant growth inhibition. In our study, many DEGs identified in *Lxx*-infected plants were associated with sugarcane cell wall degradation and this effect was more pronounced in stalks [14].

The molecular mechanism(s) regulating the response to various pathogenic bacterial infections may be similar among different crops as the biological processes underpinning growth and development remain same across plant species. Common response of secondary metabolism was observed in sugarcane during *Acidovorax avenae* subsp. *avenae* [34] and *Colletotrichum falcatum* [35] infection. The activity of genes involved in these two important pathways was also found altered in this study, suggesting the shared molecular response of sugarcane to diverse bacterial pathogenesis.

## Conclusion

In conclusion, the present study has considerably advanced the molecular understanding of RSD in sugarcane. Transcriptome sequencing of sugarcane leaves and stalks during *Lxx* infection identified a total of 107,750 unigenes, with nearly 60% of them were longer than 1,000 bp. Gene expression analysis detected 38,513 transcripts representing both RSD-affected and healthy plants studied. Functional analysis revealed that the DEGs are mainly involved in “metabolic pathways”, “signal transduction”, and “plant–pathogen interactions”. *Lxx* infection caused significant variation in transcript abundance associated with chloroplast function. *Lxx* infection inhibited gibberellin response through up-regulation of DELLA and down-regulation of GID1 proteins. The alteration of GA response and the chloroplast-associated gene expression may account for the majority of growth retardation observed in RSD-affected plants. A number of DEGs were associated with plant-pathogen interactions such as those involved in secondary metabolism, protein phosphorylation/dephosphorylation, cell wall biosynthesis, and phagosomes, implicating an active defense response to *Lxx* infection. A better understanding of the molecular and physiological basis of RSD pathogenesis-related responses will help develop tools and technologies for producing RSD-resistant sugarcane varieties through conventional and/or molecular breeding.

## Supporting information

S1 Fig Length distribution of the assembled sugarcane transcripts and unigenes. (PDF)

S2 Fig Results of transcriptome assembly evaluation results by BUSCO. (PDF)

S3 Fig Distribution of normalizedexpression of transcripts detected in all samples. (PDF)

S4 Fig Heat map of correlations among replicates based on the gene expression profile. (PDF)

S1 File Primers used for RT-qPCR analysis of differentially expressed genes. (PDF)

S2 File Differential expression analysis of all genes in *Lxx*-infected compared to healthy sugarcane. (XLSX)

## Abbreviation

ABA: Abscisic acid
CAT: Catalase
DEG: Differentially expression gene
ET: Ethylene
FPKM: Fragments per kilobase of transcript per million mapped reads
GA: Gibberellic acid
GAPDH: Glyceraldehyde-3-phosphate dehydrogenase
GO: Gene ontology
HL: Healthy leaves
HS: Healthy stalks
IAA: Indoleacetic acid
IL: Infected leaves
IS: Infected stalks
JA: jasmonic acid
KEGG: Kyoto encyclopedia of genes and genomes
KOG: EuKaryotic Orthologous Group
Log_2_FC: Log_2_ fold change
Lxx: *Leifsonia xyli* subsp. *xyli*
NR: NCBI non-redundant database
POD: Peroxidase
RNA-seq: RNA sequencing
RT-qPCR: Reverse transcription quantitative real-time polymerase chain reaction
RSD: Ratoon Stunting Disease
SOD: Superoxide dismutase
TF: Transcription factor
