# Supplementary figures and images for: A transcriptomic analysis of sugarcane response to Leifsonia xyli subsp. xyli infection
Source: PLoS One. 2021 Feb 2;16(2):e0245613. doi: 10.1371/journal.pone.0245613 (PMC7853508; doi:10.1371/journal.pone.0245613)

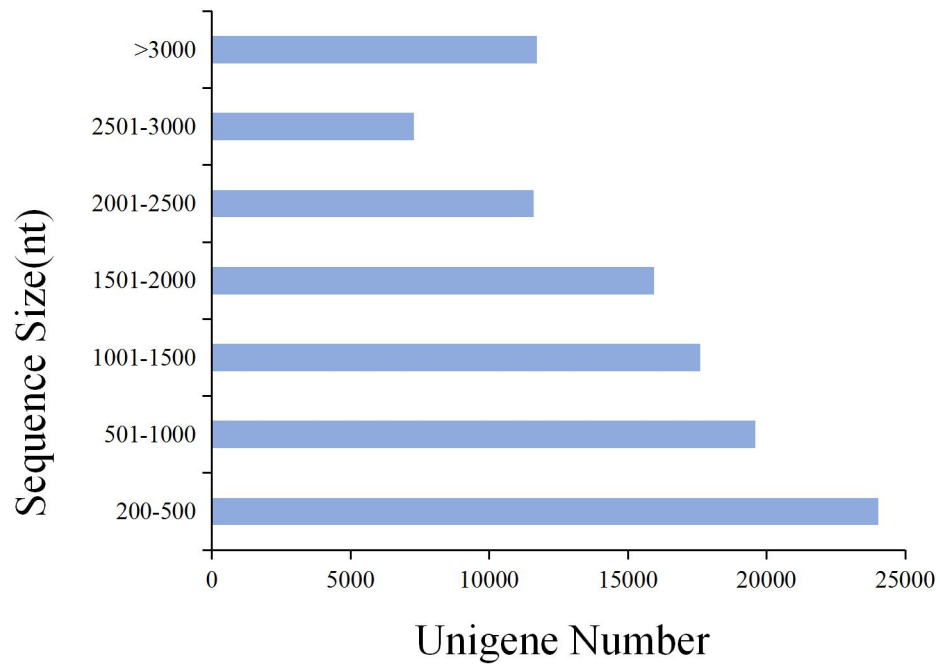

**S1 Fig.** Length distribution of the assembled sugarcane transcripts and unigenes

Supplement: S1 Fig — (PDF) [file pone.0245613.s001.pdf]

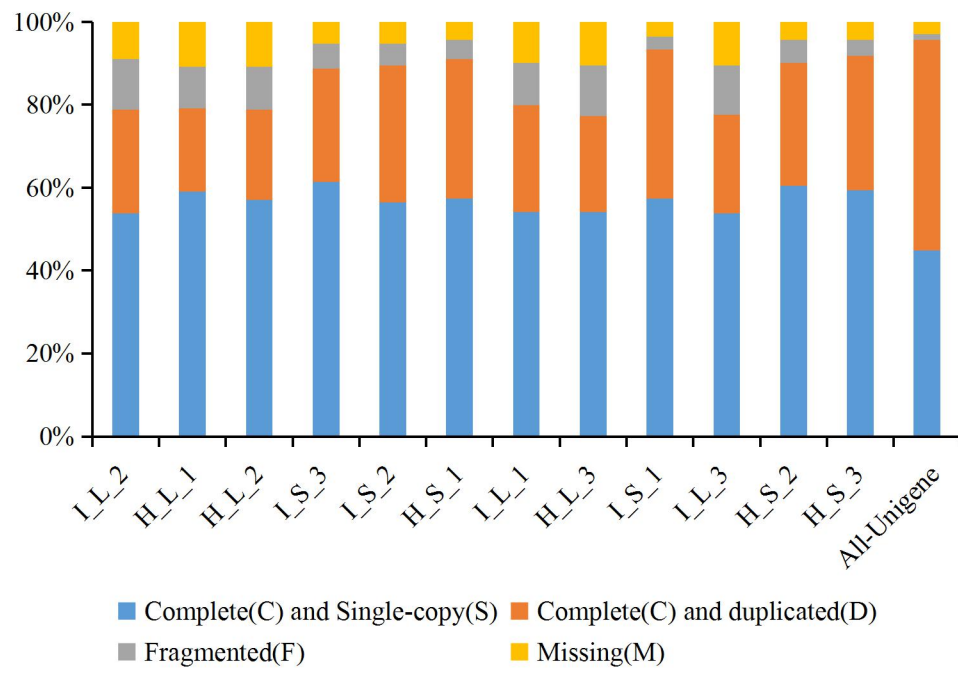

**S2 Fig.** Results of transcriptome assembly evaluation results by BUSCO.

Supplement: S2 Fig — (PDF) [file pone.0245613.s002.pdf]

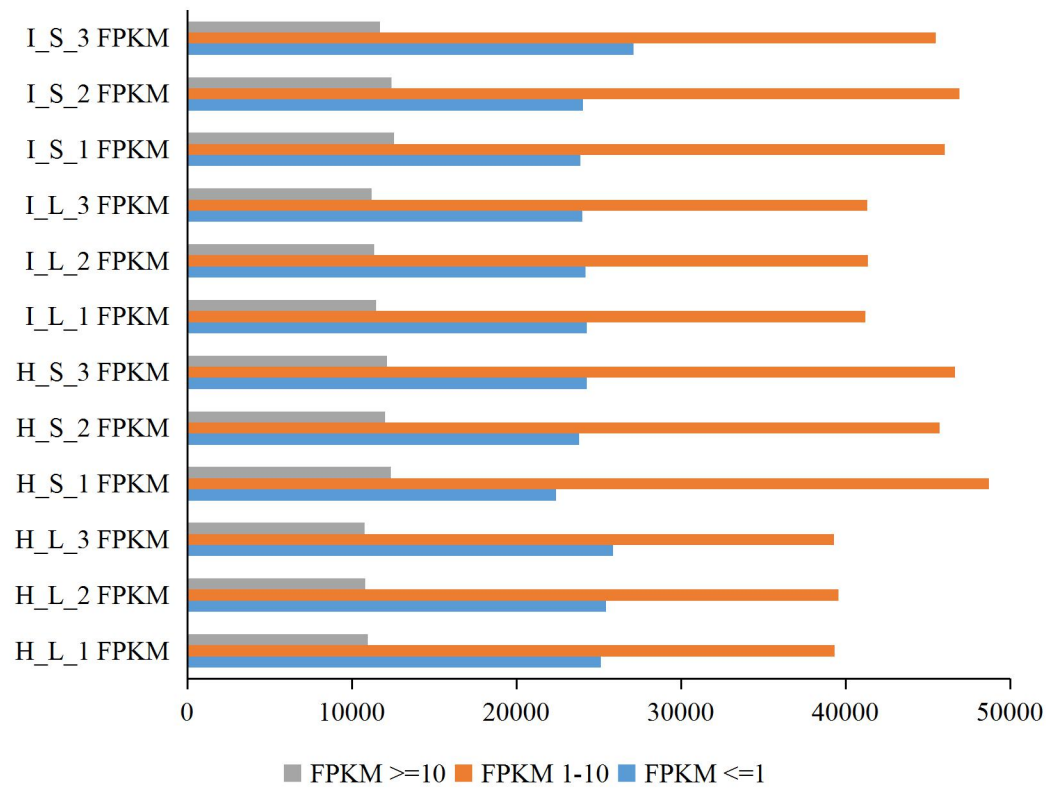

**S3 Fig.** Distribution of normalized expression of transcripts detected in all samples.

Supplement: S3 Fig — (PDF) [file pone.0245613.s003.pdf]

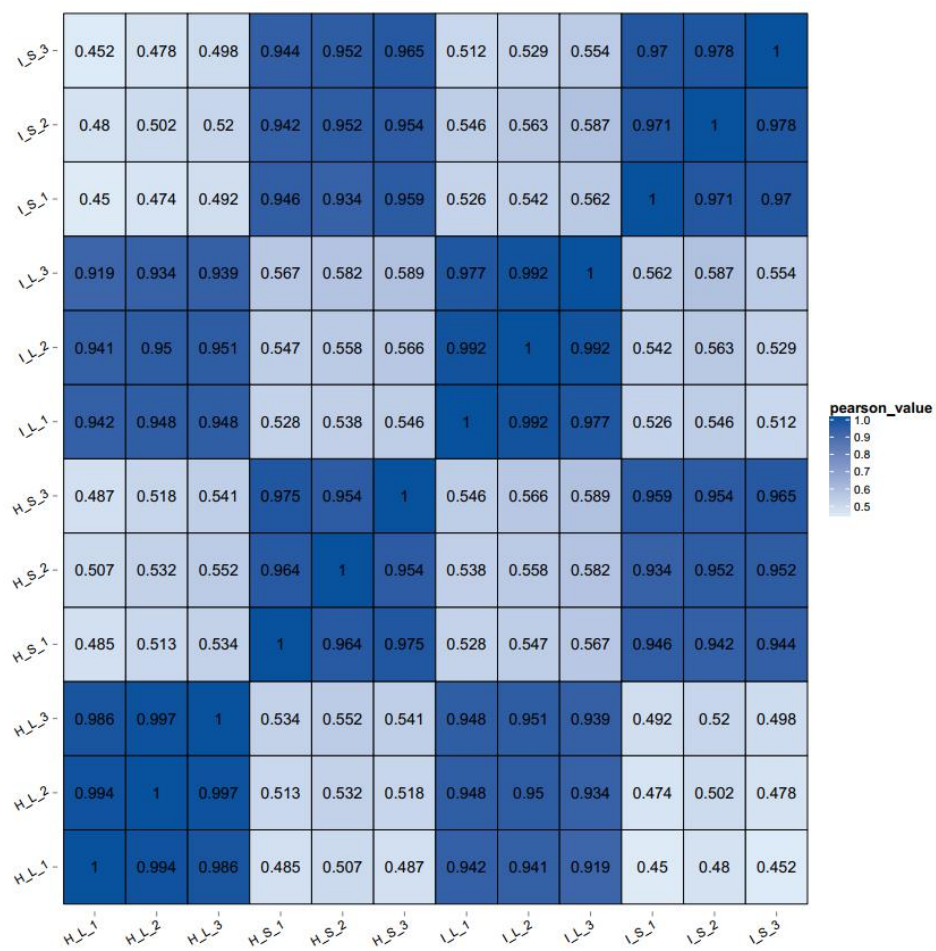

**S4 Fig.** Heat map of correlations among replicates based on the gene expression profile.

Supplement: S4 Fig — (PDF) [file pone.0245613.s004.pdf]
