# Supplementary material for: A transcriptomic analysis of sugarcane response to Leifsonia xyli subsp. xyli infection
Source: PLoS One. 2021 Feb 2;16(2):e0245613. doi: 10.1371/journal.pone.0245613 (PMC7853508; doi:10.1371/journal.pone.0245613)
Supplement: S1 File — (PDF) [file pone.0245613.s005.pdf]

S1 File. Primers used for RT-qPCR analysis of differentially expressed genes.

Gene_id	Sequence length	Pair rating	Product Tm	Forward_primer	Reverse_primer	Description
Unigene11031_All	1191	246	53	CCACTTAGAATAGCCAAACAG	TACCAGGCAATCCGAAACT	envelope membrane protein (chloroplast)
Unigene30239_All	1157	259	54	AAAGAACCACTTAGAATAGCCA	GGTGGAATACCAGGCAATC	chlorophyll a-b binding protein M9, chloroplastic
Unigene10678_All	1726	206	53	TGTCCGAAGATGAGATTGC	AGAAAAAACCTGAAAACAAAA	calcium-dependent protein kinase 17 isoform X2
Unigene41543_All	1796	221	55	ACTGGCGACAGCAATAACA	AAACATCAACCACGGCAAA	hypothetical protein SORBI_3002G231300
Unigene7405_All	983	236	54	TGGAGTGATGGGAGTAGAGC	TGACTATGCCCTGTAAGACC	RNA-binding (RRM/RBD/RNP motifs) family protein
Unigene115_All	6340	524	52	AAAAACTGTTGCTACTAACTGAA	AACAAGTCAAGGAGCACATT	MACPF domain-containing protein NSL1
Unigene36011_All	459	255	55	TATCAAAGAGAGAGGAGACATGC	CAACTGGCAGAGCAACGC	Putative expansin-A17
Unigene39498_All	546	234	56	CGATAAGACACGAGACCGC	GCGAGAGGAAGAAGAAGCG	xyloglucan endotransglucosylase/hydrolase protein 8
Unigene34604_All	1886	250	52	CACTGGAGATCGTGACATT	AAAGTTCAGAGTGCGATAAA	polyprotein
